# Cell-state transitions and microenvironmental remodeling in thyroid cancer progression revealed by single-cell and spatial transcriptomics

**DOI:** 10.3389/fimmu.2026.1904196

**Published:** 2026-07-08

**Authors:** Xin Wang, Deshuang Tao, Jinming Xu, Wensi Liu, Zhiwu Ji, Hairui Zhou, Zhen Wang

**Affiliations:** 1 Department of General Surgery, First Affiliated Hospital of Jiamusi University, Jiamusi, Heilongjiang, China; 2 Department of Epilepsy, Jiamusi Central Hospital, Jiamusi, Heilongjiang, China; 3 Department of General Surgery, Jiamusi Central Hospital, Jiamusi, Heilongjiang, China; 4 Department of Nephrology, First Affiliated Hospital of Jiamusi University, Jiamusi, Heilongjiang, China; 5 Key Laboratory of Microecology-immune Regulatory Network and Related Diseases School of Basic Medicine, Jiamusi University, Jiamusi, Heilongjiang, China

**Keywords:** anaplastic thyroid carcinoma, papillary thyroid carcinoma, single-cell RNA sequencing, spatial transcriptomics, thyroid cancer, tumor microenvironment

## Abstract

Thyroid cancer ranges from indolent differentiated tumors to metastatic, radioiodine-refractory and anaplastic disease. Conventional histology and molecular classification define major risk groups but do not resolve the cell states and spatial heterogeneity that accompany progression. This review integrates recent single-cell RNA sequencing, spatial transcriptomics and validation studies across localized papillary thyroid carcinoma (PTC), metastatic or radioiodine-refractory differentiated thyroid cancer (DTC), poorly differentiated thyroid carcinoma (PDTC) and anaplastic thyroid carcinoma (ATC). Current evidence indicates that progression is accompanied by changes in malignant epithelial states, stromal regions and immune patterns. Malignant epithelial cells shift from follicular-like programs toward partial EMT-like, dedifferentiation-like and anaplastic states; stromal changes include invasive borders, stiff peritumoral matrix and CAF/ECM-rich poorly differentiated regions; and immune patterns differ between progressive PTC, indolent lymphoid-organized tumors and myeloid-rich ATC. Functionally supported examples, including POSTN-IL-4 signaling, CCL20/CXCL5 macrophage-tumor reciprocal signals and SIGLEC15-associated checkpoint signaling, show how these technologies can generate therapeutic hypotheses. Yet most datasets are cross-sectional, and many ligand-receptor interactions remain computational candidates. We use the available evidence to relate epithelial plasticity, genomic context, stromal regions, immune remodeling and candidate cell-cell interactions across disease states, while separating observed associations from established stepwise tumor evolution. This synthesis highlights mechanisms and therapeutic hypotheses that require functional and clinical validation.

## Highlights

- Single-cell studies resolve malignant states across thyroid cancer progression.
- Spatial data place CAF/ECM programs at invasive and poorly differentiated regions.
- Immune patterns differ between progressive PTC, indolent PTC and ATC.
- Candidate cell-cell interactions are graded by transcript, spatial, protein and functional support.

## Introduction

Thyroid cancer presents across a broad clinical range, and its behavior is closely linked to differentiation status. Most papillary and follicular thyroid carcinomas retain differentiated features and are controlled with surgery, radioactive iodine and thyroid-stimulating hormone suppression (1, 2). The greatest clinical challenges arise in recurrent or metastatic differentiated thyroid cancer, poorly differentiated thyroid carcinoma and anaplastic thyroid carcinoma, where loss of differentiation is accompanied by invasive behavior, radioiodine resistance and disease-related mortality (3–5). Understanding how differentiated tumors move toward these aggressive phenotypes has become a major theme in thyroid cancer biology.

Low-risk PTC and papillary thyroid microcarcinoma (PTMC) raise a distinct clinical management question. Many such tumors grow slowly, and selected patients who meet appropriate clinical and ultrasonographic criteria may be followed with active surveillance without immediate surgery (6, 7). NIFTP reflects a related concern: noninvasive, very-low-risk follicular-patterned tumors should not be overdiagnosed or overtreated (8). Accordingly, this review does not examine the clinical management of low-risk thyroid nodules or low-risk PTC in detail. The focus is whether single-cell and spatial transcriptomic studies can help identify cell states and tissue regions associated with lymph-node metastasis, radioiodine refractoriness, dedifferentiation and anaplastic transformation (9).

Histology, molecular classification and bulk transcriptomics have clarified major risk groups in thyroid cancer, but they provide limited resolution of the cellular programs and tissue regions that accompany progression (10–12). Bulk transcriptomic profiles add pathway-level information, yet they blend signals from malignant epithelial cells, fibroblasts, endothelial cells and immune populations. The newer single-cell and spatial literature is also unevenly distributed across disease states: most data come from PTC, metastatic or radioiodine-refractory DTC and ATC, with fewer studies focused on FTC, PDTC or medullary thyroid carcinoma as independent entities. Many studies now describe individual cell states, markers or interaction candidates; fewer synthesize them along the disease course from localized PTC to metastatic, radioiodine-refractory and anaplastic states.

Single-cell RNA sequencing identifies malignant, stromal and immune cell states, and spatial transcriptomics shows where these states are positioned within tumor tissue. Foundational studies have connected localized PTC, lymph-node metastasis and radioiodine-refractory disease with follicular-like, partial-EMT-like and dedifferentiation-like malignant states (13). Studies of DTC-to-ATC transformation and spatial progression extend this pattern to ATC-associated inflammatory, mesenchymal, CAF/ECM-rich and immune-suppressive regions (14–16). Spatial studies of dedifferentiated thyroid carcinoma further connect low-differentiation or ATC regions with angiogenesis, extracellular matrix activity and suppressive immune neighborhoods (17, 18).

**Figure 1:**
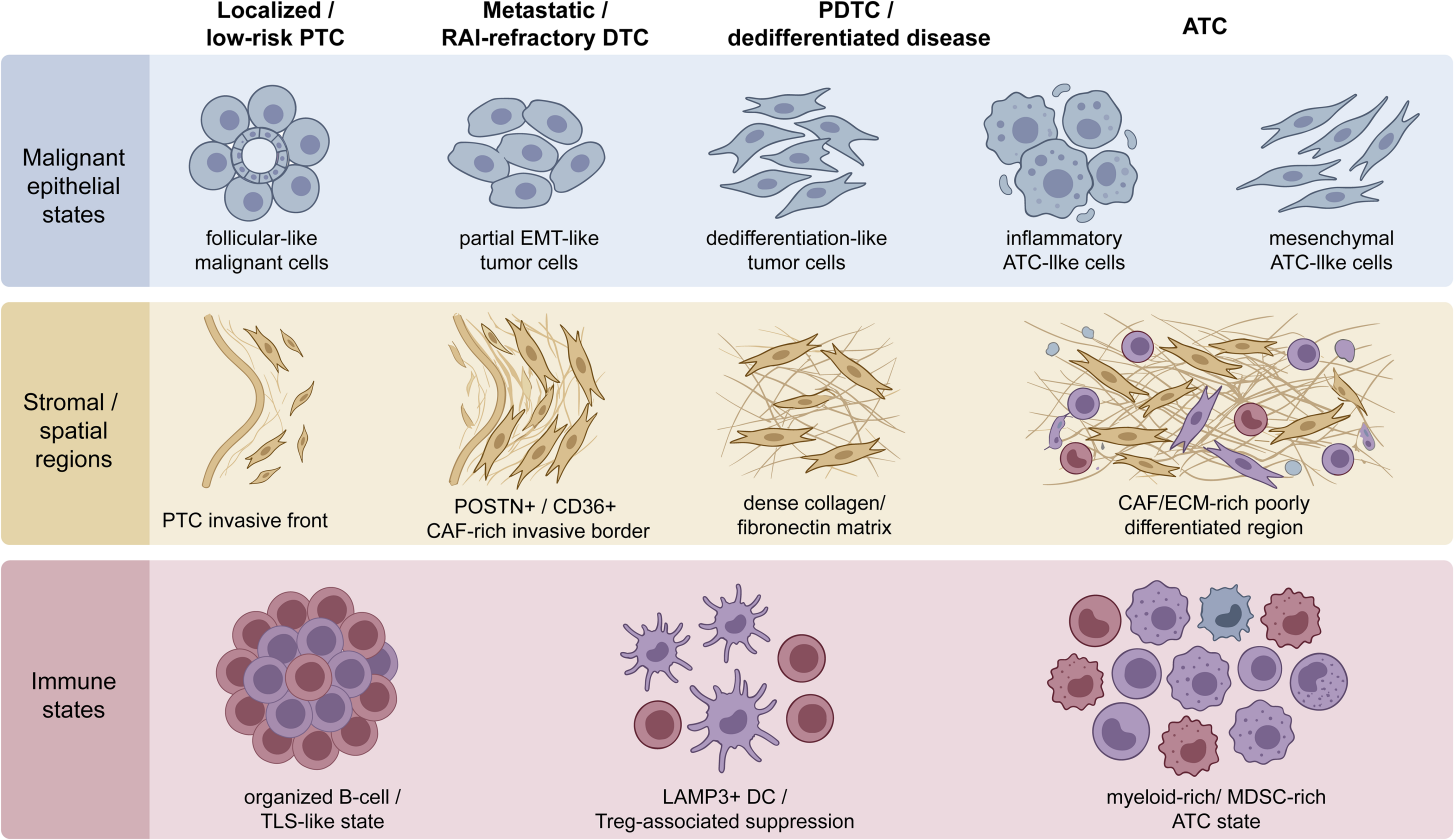
Disease-state-associated cellular patterns in thyroid cancer single-cell and spatial studies. The columns organize findings across localized or low-risk PTC, metastatic or radioiodine-refractory DTC, PDTC or dedifferentiated disease, and ATC. The rows summarize malignant epithelial states, stromal or spatial regions, and immune-state patterns reported across current single-cell and spatial studies. The schematic is intended as a summary of disease-state associations rather than a uniform path followed by individual tumors.

This review focuses on how single-cell and spatial transcriptomic studies inform the interpretation of thyroid cancer progression. It discusses recurring epithelial states, stromal regions and immune changes across localized PTC, metastatic or radioiodine-refractory DTC, PDTC and ATC. **Figure 1** summarizes these disease-state-associated cellular patterns. It further evaluates how these changes relate to lymph-node metastasis, radioiodine refractoriness, dedifferentiation and anaplastic transformation, with attention to evidence from spatial localization, protein validation, functional experiments and clinical association.

## Methods and search strategy

PubMed was searched for English-language articles published from 2015 to June 2026. Search keywords covered thyroid cancer subtypes, including thyroid cancer, papillary thyroid carcinoma, follicular thyroid carcinoma, poorly differentiated thyroid carcinoma and anaplastic thyroid carcinoma; single-cell and spatial technologies, including single-cell RNA sequencing, single-nucleus RNA sequencing, spatial transcriptomics, spatial omics, Visium, GeoMx and CosMx; and clinically or mechanistically relevant topics, including low-risk papillary thyroid microcarcinoma, active surveillance, NIFTP, PDTC/ATC genomics, pediatric thyroid cancer, ATC therapeutic targets, BRAF/MEK inhibitor resistance, epigenetic regulation and methodological issues in scRNA-seq, ST and ligand-receptor analysis. The thyroid cancer single-cell and spatial transcriptomics search retrieved 248 records. Original thyroid cancer scRNA-seq and ST studies were prioritized. Functional studies, genomic and proteogenomic analyses, clinical consensus articles and methodological papers were included when they helped interpret cell states, spatial localization or candidate cell-cell interactions. Findings were discussed according to study type and level of support, including transcript-level inference, spatial localization, protein validation, functional experiments and clinical association (19, 20). **Supplementary Table 1** summarizes the main original single-cell, spatial and functional studies and their subtype coverage.

## Malignant epithelial state changes during progression

Current single-cell and spatial studies point to three recurring epithelial changes in thyroid cancer progression. Differentiated or localized PTC usually retains follicular-like tumor-cell states and thyroid identity programs (13, 14). Metastatic or RAI-refractory lesions more often contain partial-EMT-like, proliferative, stress-adapted or dedifferentiation-like cells (13, 14, 21, 22). PDTC, ATC and coexisting high-grade regions show stronger loss of thyroid identity, with inflammatory, mesenchymal or extracellular-matrix-related tumor-cell programs (17, 18, 23). These observations should be weighed by the support available in each study: spatial localization and perturbation experiments provide stronger support than reanalysis alone or very small cohorts.

Genomic and proteogenomic studies help explain why these states appear with progression. BRAF-driven MAPK signaling is a common driver in PTC (24, 25). Progression toward PDTC or ATC is usually accompanied by additional events, including TERT promoter activation, TP53 loss, PI3K-pathway alterations, chromosomal instability, higher mutational burden and clonal evolution (24, 26). Recent proteogenomic profiling of PDTC and ATC links these changes to DNA repair, immune-cell infiltration, ribosome biogenesis and myeloid-rich therapeutic subtypes (27). Single-cell studies rarely assign a given state to a mutation within the same tumor. The genomic data help connect thyroid-identity loss and epithelial plasticity with accumulated driver alterations, beyond marker-level transcriptional shifts.

Partial EMT is one of the best-supported malignant-cell programs in this literature. In an adult-onset BRAF V600E mouse model of PTC, single-cell analysis resolved malignant thyrocyte subpopulations with different degrees of mesenchymal transformation and placed them along an EMT trajectory from intermediate states to more mesenchymal and malignant states (23). Organoid experiments supported the EMT-related phenotype of these subpopulations, and p53 loss increased malignant potential through metabolic plasticity. In human PTC, consensus non-negative matrix factorization of malignant cells identified an EMT-related gene expression program, GEP3, associated with lymph-node metastasis and poor clinical outcome (21). Within GEP3-high cells, ELF3 was nominated as a hub gene, and ELF3 knockdown reduced EMT markers, migration and invasion in PTC cell models. These findings support partial EMT as a recurrent malignant thyrocyte state, especially in BRAF-mutant and metastatic PTC.

Metastatic PTC contains additional tumor-cell states that overlap with, but are not limited to, partial EMT. One study integrating single-cell RNA sequencing, spatial transcriptomics and functional assays identified an APOE-low or APOE-negative tumor-cell population associated with advanced stage and cervical lymph-node metastasis (22). APOE expression decreased in later pseudotime states and was lower in tumor cells from lateral cervical lymph-node metastases than in central neck metastases; APOE overexpression reduced PTC-cell proliferation and invasion *in vitro* and suppressed tumor growth in xenograft models. A separate single-cell study of six PTC tumors found that malignant cells from lymphatic-metastasis cases showed higher activity of pathways related to proliferation, migration and survival (28). Another analysis of primary and metastatic PTC samples used Scissor to define metastasis-associated epithelial cells with increased VEGF, androgen and TGF-beta pathway activity (29). These studies indicate that metastatic PTC is accompanied by several invasive, proliferative or stress-adapted epithelial states, although individual markers remain study-specific.

Loss of thyroid differentiation and radioiodine resistance form another recurrent pattern, consistent with the central role of NIS in iodide handling and with clinical mechanisms of radioiodine resistance (30, 31). In the multi-site PTC single-cell study discussed above, dedifferentiation-like malignant thyrocytes were more common in radioiodine-refractory distant metastases (13). Mechanistic studies add detail to this observation. In BRAF-mutated radioactive iodine-refractory PTC cells, NOX4-derived oxidative DNA damage recruited OGG1 and MSH2/MSH6 with DNMT1, impaired chromatin access of PAX8 and NKX2.1 and suppressed NIS expression (32). Another study used single-cell data from 28 thyroid cancer or adjacent normal tissues to trace ATM expression during dedifferentiation and found stepwise ATM upregulation during progression, with higher ATM expression in ATC and radioiodine-refractory tumors (33). These data connect dedifferentiation-like malignant states with reduced iodide-handling gene expression and increased DNA-damage tolerance.

At the poorly differentiated and anaplastic end of thyroid cancer, malignant epithelial states lose thyroid identity and acquire inflammatory, mesenchymal or ATC-like programs. Genomic studies of PDTC and ATC show accumulated driver alterations and clonal evolution during dedifferentiation (24, 26). Integrated analysis of thyroid cancer dedifferentiation also supports a shift from differentiated PTC-like tumors toward ATC-like programs (34). TERT biology is relevant to this transition; experimental BRAF-mutant models show that TERT can accelerate dedifferentiation, and thyroid-cancer reviews place TERT activation among key progression events (35, 36). Lu et al. integrated single-cell transcriptomic and genetic data to order epithelial states from normal thyroid follicular cells and PTC cells toward inflammatory ATC and mesenchymal ATC states (14). Inflammatory ATC cells retained epithelial features with inflammatory programs, while mesenchymal ATC cells lost thyroid differentiation genes and expressed mesenchymal, collagen and extracellular-matrix programs. A spatial transcriptomic study of tumors containing coexisting DTC, high-grade follicular-cell-derived carcinoma, PDTC or ATC regions also showed decreasing thyroid differentiation markers toward ATC regions and identified SERPINE1+ and KRT5+ ATC regions (17). Relapsed follicular thyroid carcinoma adds another piece to this pattern. FTC genomics is less represented than PTC, although whole-genome sequencing has identified recurrent DGCR8 alterations in FTC (37). Across 46, 739 cells from PTC, follicular-variant PTC, relapsed FTC and ATC, single-cell analysis identified ATC-like cells in relapsed FTC, marked by high UBE2C expression and ATC molecular characteristics; UBE2C was functionally linked to FTC proliferation, xenograft growth and metabolic changes (38). This evidence is limited by the small number of tumor specimens, but it broadens the discussion of ATC-like states beyond PTC-derived transformation models.

Taken together, the epithelial literature is strongest on three points. Differentiated tumors contain follicular-like states, metastatic or RAI-refractory tumors add invasive, proliferative, DNA-damage-tolerant or dedifferentiation-like states, and ATC contains inflammatory or mesenchymal states with marked loss of thyroid identity. Functional evidence strengthens selected observations, including EMT regulation by ELF3, APOE-associated restraint of PTC growth and invasion, and UBE2C-driven growth in relapsed FTC. The weaker areas are timing and breadth: most datasets are cross-sectional, FTC and PDTC are under-sampled, and some states come from small cohorts or public-data reanalysis.

## Spatial stromal regions in invasion and dedifferentiation

**Figure 2:**
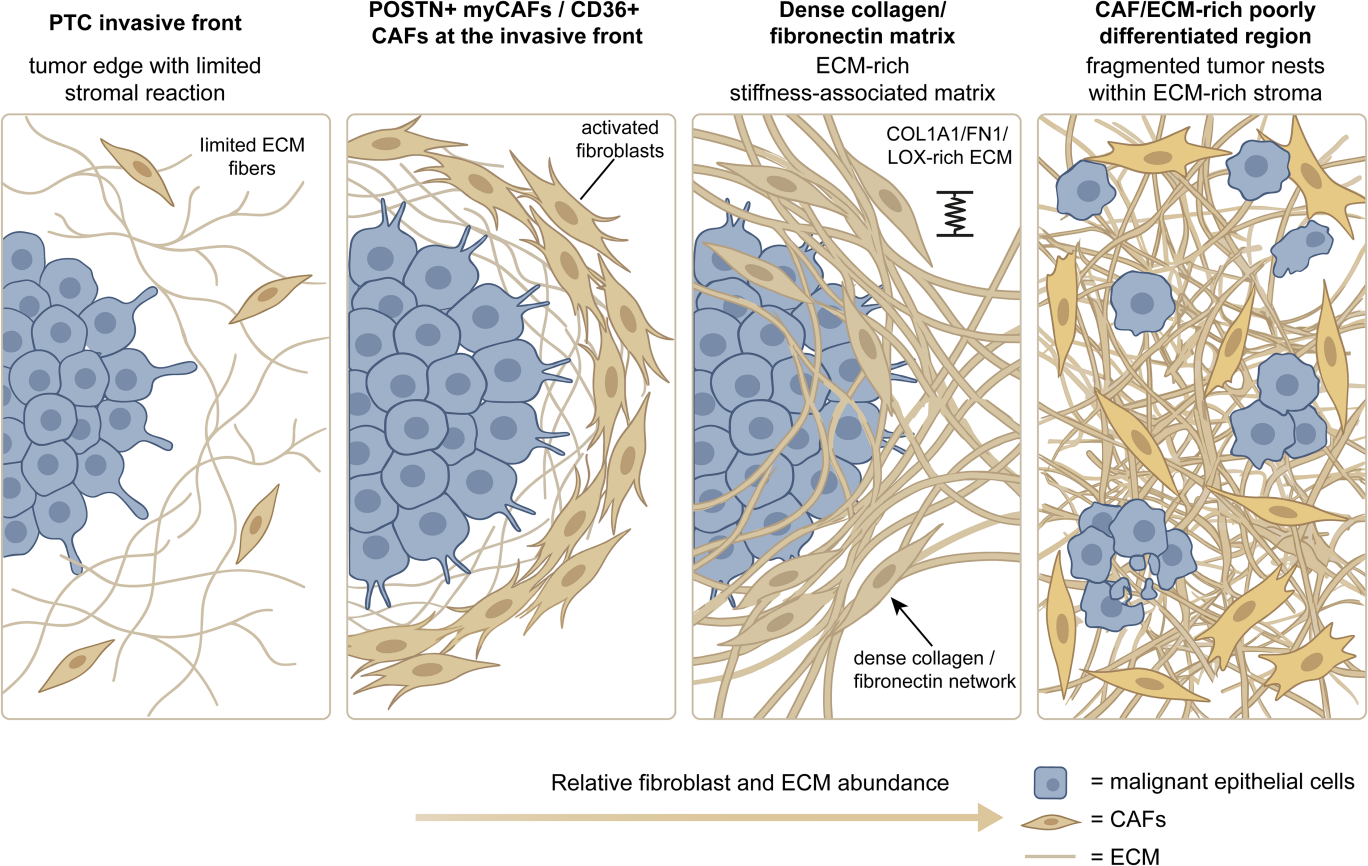
Representative stromal and extracellular-matrix-rich regions in thyroid cancer. The schematic summarizes four stromal patterns reported in single-cell, spatial and pathology-based studies: the PTC invasive front with limited stromal reaction, POSTN+ myCAF/CD36+ CAF-rich invasive fronts, dense collagen/fibronectin matrix, and CAF/ECM-rich poorly differentiated regions. The bottom scale indicates relative fibroblast and extracellular-matrix abundance across the illustrated regions.

Malignant epithelial plasticity in thyroid cancer is spatially patterned. Spatial transcriptomic studies place invasion-associated and low-differentiation programs near tumor borders, leading edges, PTC-ATC transition regions and poorly differentiated areas, with uneven distribution across tumor sections (15, 16). The same regions frequently contain higher cancer-associated fibroblast (CAF) abundance, extracellular-matrix gene expression, collagen or fibronectin-related programs and increased matrix stiffness (39–41). These observations point to four recurring tissue regions: the PTC invasive front, the stiff peritumoral matrix, the poorly differentiated or ATC-associated stroma, and boundary regions such as capsular or vascular invasion in FTC and PTC-ATC interfaces. **Figure 2** illustrates representative stromal regions with different fibroblast and extracellular-matrix abundance.

The PTC invasive front is one of the best-characterized stromal regions in the current single-cell and spatial literature. A study combining single-cell and spatial transcriptomics identified POSTN+ myofibroblastic CAFs (myCAFs) as tumor-adjacent stromal cells closely associated with invasive tumor cells, lymph-node metastasis and worse clinical outcome (16). APOD+ inflammatory CAFs in the same study were positioned farther from tumor cells and were more prominent in inflammatory stromal regions, separating tumor-adjacent matrix-producing CAFs from more distant inflammatory stromal states (16). Pathology-scale data strengthen this regional pattern: in 984 PTC cases, high fibrosis density at the invasive front was associated with lymph-node metastasis, and single-cell analysis identified metastasis-associated myoCAFs; CD36+ CAFs promoted PTC-cell migration and invasion *in vitro* (39). Earlier human-tissue work also placed CAF recruitment, collagen deposition and LOX expression at thyroid cancer invasive fronts, with LOX, COL1A1, ACTA2 and FAP patterns associated with BRAF-like tumors and lymph-node metastasis (42). These findings describe an invasive border characterized by tumor-adjacent myCAFs, collagen deposition and matrix-remodeling activity.

A second recurring region is the stiff peritumoral matrix. In a large clinical cohort, elevated peri-cancerous stiffness in PTC was linked to lymph-node metastasis, recurrence and mortality risk; multiplex immunofluorescence connected this stiffness pattern with alpha-SMA, PDGFR-alpha, p-MLC2 and COL-I deposition in peri-cancerous regions (40). FN1 and FN-binding integrin subunits showed higher expression in BRAF V600E-positive PTC, and high FN1 expression correlated with lymph-node metastasis, extrathyroidal extension and recurrence risk (43). CAF-derived periostin promoted PTC growth in functional models, supporting the biological relevance of POSTN-rich CAFs in matrix-rich PTC stroma (44). In ATC models, collagen-coated high-stiffness hydrogels increased proliferation, clonogenicity, migration and invasion, activated RhoA/Rac1/Cdc42 and integrin alpha6beta4-FAK signaling, and FAK inhibition reduced stiffness-dependent tumor-promoting effects (41). These studies do not prove one continuous sequence from CAF activation to metastasis, but they place collagen deposition, fibronectin-integrin signaling, tissue stiffness and FAK activation within the same matrix-mechanics process.

The stromal region associated with low differentiation is more prominent in PDTC and ATC. A spatial transcriptomic series spanning paratumor tissue, PTC, locally advanced PTC and ATC showed stage-specific remodeling of tumor leading edges and identified SERPINE1+ fibroblasts whose abundance and SERPINE1 expression increased with malignant progression and were associated with prognosis in ATC (15). Another spatial study across PTC, FTC, PDTC and ATC found that thyroid differentiation score varied within tumor sections and was inversely correlated with fibroblast density, with ATC showing the lowest differentiation scores (18). A Visium-based analysis of PTC, PDTC and ATC specimens further showed that CAFs were more abundant in poorly differentiated regions and exhibited increased glycolytic activity; CAF-conditioned medium promoted PTC proliferation, invasion and dedifferentiation while reducing radioiodine uptake in co-culture models (45). In this region, the spatial signal is the co-occurrence of low differentiation, increased fibroblast abundance, SERPINE1+ fibroblasts and altered CAF metabolism.

High-grade and boundary regions provide additional support. ATC studies connect high-grade tumor areas with ECM-related pathways, collagen deposition, fibroblast activation and invasion-associated markers such as COL7A1 and LAMC2 (46, 47). Spatial immunofluorescence across a PTC-ATC boundary showed increased alpha-SMA CAFs, collagen III/VI, TGFBI and YAP signal toward ATC regions (48). FTC evidence remains sparse, but a single-case spatial transcriptomic study found higher ECM and EMT-related gene expression at capsular and vascular invasion regions, with POSTN and DPYSL3 increasing toward the tumor periphery (49). These data broaden the regional pattern beyond conventional PTC, while their small cohorts and single-case designs limit how far the pattern can be generalized.

Across the available studies, thyroid cancer progression is accompanied by recurring stromal regions, with no evidence for a uniform stromal change across the whole tumor. In PTC, the invasive front and stiff peritumoral matrix are the best-supported regions. In PDTC and ATC, low-differentiation regions are accompanied by increased fibroblast abundance, SERPINE1+ fibroblasts, glycolytic CAF activity and stronger matrix-related signaling. FTC and PTC-ATC boundary studies add useful spatial detail; broader sampling is needed to determine whether these observations extend across thyroid cancer subtypes. This regional view keeps stromal involvement in invasion and dedifferentiation distinct from immune remodeling and specific cell-cell signaling.

## Immune-state shifts in metastatic and dedifferentiated thyroid cancer

Single-cell and spatial studies indicate that immune composition varies with clinical behavior in thyroid cancer. Indolent PTC, metastatic or progressive PTC and ATC differ in antigen-presentation capacity, lymphoid organization, myeloid-cell abundance and T-cell functional state. A large integrated single-cell analysis of 405, 077 cells from thyroid cancer and normal thyroid samples also underscored intertumoral differences in immune, endothelial and mesenchymal cell populations across PTC, ATC and metastatic disease (50). The strongest evidence supports three recurring immune patterns: LAMP3+ dendritic-cell and Treg-associated suppression in progressive PTC, B-cell and tertiary-lymphoid-structure (TLS)-like signals in indolent PTC or immunotherapy-sensitive contexts, and myeloid-cell accumulation with CD8+ T-cell dysfunction in dedifferentiated or anaplastic disease.

Age is an important modifier of thyroid cancer biology and should not be collapsed into the adult PTC progression pattern. Pediatric and children/adolescent-young-adult PTCs often show a different driver profile from adult tumors, with a prominent role for fusion oncogenes such as RET and NTRK rearrangements and a lower frequency of TERT promoter mutations. Recent single-cell studies extend this distinction to the tumor microenvironment. In CAYA-PTC, CD4 T follicular helper-like and exhausted CD8 T-cell populations were more prevalent than in adult PTC, and malignant thyrocytes showed differentiation changes that differed from adult tumors. Another age-stratified single-cell analysis suggested age-dependent epithelial reprogramming and different immune/TAM states between younger and adult patients. These findings indicate that pediatric or young-adult PTC may reach invasive or metastatic phenotypes with cellular and immune changes that differ from adult BRAF-like PTC. The available cohorts remain small, so pediatric CAF and immune differences are best treated as subgroup modifiers, without assuming one pattern that applies to all pediatric or young-adult tumors (51–54).

Progressive PTC shows early evidence of impaired immune surveillance. In a single-cell study of 151, 238 cells from 18 adjacent, non-progressive PTC and progressive PTC specimens, progressive tumors contained fewer overall immune cells, showed enhanced tumor-cell immune evasion and had disrupted antigen-presentation function (55). This study identified LAMP3+ dendritic cells as a population more common in progressive PTC and associated with advanced T stage and poor prognosis. These cells were linked to CD8+ T-cell exhaustion and Treg recruitment, and the findings were supported by spatial transcriptomics, immunohistochemistry, multiplex immunohistochemistry and bulk transcriptomic validation (55).

Metastatic PTC adds a stronger myeloid and regulatory component to this immune pattern. A single-cell analysis comparing metastatic PTC, adjacent tissue and non-metastatic PTC found that metastatic disease was associated with M2-like macrophages, conventional type 2 dendritic cells and Tregs, while monocytes and B cells were described as potentially favorable immune populations (56). Another study profiled primary tumor, adjacent thyroid and lymph-node metastasis from a young patient with PTC and validated selected findings in 61 tumors; malignant epithelial cells were proposed to suppress macrophage activity through MIF pathway signaling, and CD74 expression was higher in tumors with lymph-node metastasis (57). In six PTC tumors with or without lymphatic metastasis, CD8+ resident memory T cells were highlighted as regulators of lymphatic metastasis, with changes in antigen-presentation and T-cell signaling markers (28). Bilateral PTC data also show that malignant, immune and stromal cell states can differ between tumors from the same patient context (58). Multiplex immunofluorescence in 17 DTC samples further showed higher intratumoral PD-1 and PD-L1 expression than adjacent thyroid tissue, with distant metastatic cases showing trends toward more Tregs and M2 macrophages and fewer CD8+ T cells and M1 macrophages (59). These studies differ in design and scale, but they consistently place metastatic PTC near immune suppression, altered antigen presentation and macrophage/T-cell state changes.

PTC also contains immune states associated with indolent behavior. In early-stage PTC, single-cell profiling of 10 tumors with validation in 25 additional tumors found that tumor-infiltrating B cells, particularly germinal-center B cells, were more abundant in indolent tumors (60). These B cells formed TLS-like clusters, showed increased proliferation in indolent cases and suppressed thyroid-cell proliferation in experimental assays. This separates immune infiltration linked to tumor control from immune infiltration linked to progression. Hashimoto thyroiditis-associated PTC adds another immune context: single-cell studies report altered B-cell, plasma-cell, myeloid and T-cell states in HT-PTC and in inflammation-to-cancer comparisons (61–63). A related observation appears in ATC: a single-cell study comparing ATC and PTC found CXCL13+ T lymphocytes were more common in ATC samples and associated them with early TLS-like structures, which may help explain why ATC can show greater sensitivity to immunotherapy than late-stage PTC despite its aggressive biology (64).

Loss of thyroid differentiation is accompanied by increased myeloid-cell involvement. In a spatial transcriptomic study of 12 PTC, FTC, PDTC and ATC samples, dedifferentiation was associated with increased myeloid cells and CAFs, together with reduced NK cells and endothelial cells (18). Within tumor sections, thyroid differentiation score correlated spatially with myeloid-cell density, and MDSC score was negatively correlated with thyroid differentiation score. JAK-STAT and VEGF pathway activity were also associated with myeloid-cell infiltration in dedifferentiated tumors. A spatial study of seven tumors containing coexisting ATC, PDTC and DTC regions found that ATC regions showed stronger immune-suppression, angiogenesis and extracellular-matrix programs, with TYMP+ tumor-associated macrophages concentrated in ATC areas (17). Spatial analysis of PTC, ATC, ATC-associated lymphatic metastasis and rare gastric metastasis added a related observation: SFRP4-high tumor-specific myeloid cells correlated with dedifferentiation and poor prognosis and localized close to CD44+ tissue-stem-like cells (65).

ATC appears immunologically active but functionally constrained. In the ATC/PTC single-cell study, multiple immune-cell types formed a more immunosuppressive phenotype in ATC than in PTC, while cytotoxic CD8+ effector T cells and NK cells were more frequent in PTC samples (64). An independent integrative single-cell analysis of ATC similarly reported an immunosuppressive state with exhausted T-cell and myeloid programs (66). A later analysis of two ATC and two PTC single-cell datasets annotated 45, 474 ATC cells and 79, 457 PTC cells and found markedly prevalent pre-exhausted CD8+ T cells in ATC datasets; it also reported 221 ATC-associated immune genes, 115 candidate biomarkers for pre-exhausted CD8+ T cells and experimental validation of GNLY by immunofluorescence and BHT101 co-incubation/flow cytometry assays (67). Prospective flow-cytometry analysis provided orthogonal clinical support: among ATC, DTC and thyroid follicular nodular disease samples, ATC showed higher MDSC frequency than DTC or nodular disease (68). These data support an ATC immune state characterized by myeloid suppression, MDSC accumulation and CD8+ T-cell dysfunction, while preserving the possibility that organized lymphoid signals may influence immunotherapy responsiveness.

**Table 1** condenses the malignant epithelial, stromal and immune findings into disease-state patterns instead of listing every marker-level observation. Three immune-state patterns emerge from the current literature. Progressive or metastatic PTC is associated with antigen-presentation dysfunction, LAMP3+ dendritic cells, Tregs, M2-like macrophages and macrophage suppression. Indolent and lymphoid-organized states contain tumor-infiltrating B cells, germinal-center B cells, TLS-like clusters or CXCL13+ T cells, suggesting that organized lymphoid responses may accompany tumor restraint or immunotherapy sensitivity in selected settings. Dedifferentiated and ATC states show myeloid-cell accumulation, reduced NK-cell signals, TYMP+ TAMs, MDSCs and CD8+ T-cell dysfunction. FTC and PDTC remain less well covered. The clearest immune patterns are antigen-presentation impairment and immune suppression in progressive or metastatic PTC, B-cell/TLS-like responses in indolent or lymphoid-organized tumors, and myeloid-cell accumulation with CD8+ T-cell dysfunction in dedifferentiated and anaplastic disease.

**Table 1:** Integrated disease-state patterns across thyroid cancer progression.

Disease-state context	Malignant epithelial pattern	Stromal/spatial pattern	Immune pattern	Interpretation and evidence limits	PMID
Low-risk or indolent differentiated disease	Retained thyroid differentiation; follicular-like malignant states where carcinoma is present; NIFTP is a low-risk boundary entity.	Invasive CAF/ECM programs are not well defined in surveillance-eligible tumors.	Indolent early PTC may show B-cell or TLS-like organization with limited dominant suppression.	Low-risk disease is a clinical-risk state; single-cell/spatial evidence for surveillance tumors remains sparse.	27078145; 32124418; 33023426; 37166389; 40069827
Localized or progressive PTC	Cancer-primed, follicular-like, partial-EMT-like and APOE-low/negative states occur across localized, invasive and metastatic PTC.	POSTN+/CD36+ CAF-rich borders, collagen/fibronectin deposition and stiff peritumoral matrix recur.	Progressive PTC shows LAMP3+ DCs, Tregs, exhausted CD8+ T cells and impaired antigen presentation.	Invasion reflects epithelial plasticity with stromal activation and immune suppression; studies are cross-sectional and markers vary.	34663816; 41480746; 38816233; 39810624; 38902966; 41606295
Metastatic or RAI-refractory DTC	Dedifferentiation-like cells and reduced iodide-handling programs increase in metastatic or RAI-refractory disease.	CAF metabolism and TGF-beta-Smad2/3 signaling link stroma to dedifferentiation and lower RAI uptake.	Metastatic PTC shows macrophage-rich/regulatory states, including MIF-CD74/CXCR4-linked suppression.	Loss of thyroid identity should be read with stromal/immune changes; treatment-response relevance needs prospective validation.	34663816; 38061122; 41761234; 41694580; 42046098
Pediatric and children/adolescent-young-adult PTC	Fusion-driven biology can differ from adult PTC; RET/NTRK rearrangements are more common and TERT promoter changes less frequent.	CAF/metabolic programs may be age-specific, including ITGA2-linked remodeling.	CAYA-PTC may show more Tfh-like CD4 T cells and exhausted CD8 T cells than adult tumors.	Age modifies driver biology and the tumor microenvironment; evidence is emerging and should remain separate from adult progression claims.	35434981; 34535459; 40719066; 42171146
PDTC, high-grade follicular-cell-derived carcinoma and ATC	Dedifferentiation features inflammatory/mesenchymal ATC programs, thyroid-identity loss and higher genomic complexity.	SERPINE1+ fibroblasts, CAF-rich low-differentiation regions, collagen/ECM programs and ATC boundaries recur.	Dedifferentiated regions show myeloid-cell accumulation, TYMP+ TAMs, MDSC signals, lower NK-cell signals and dysfunctional CD8+ T cells.	ATC combines tumor-cell reprogramming, stromal change and myeloid suppression; coexisting-region studies are small and longitudinal data limited.	37053016; 40908584; 41182416; 40234451; 42143022
FTC and other underrepresented histologies	FTC data suggest invasive peripheral programs and ATC-like states in relapsed disease.	Capsular or vascular invasion regions may show ECM/EMT programs, POSTN and DPYSL3 peripheral signals.	Immune evidence is limited compared with PTC or ATC.	FTC and PDTC remain coverage gaps; evidence is mainly single-case or small-cohort.	38280140; 38229431; 41631714

This table summarizes disease-state-associated patterns reported in published single-cell, spatial and validation studies. ATC, anaplastic thyroid carcinoma; CAF, cancer-associated fibroblast; CAYA, children/adolescent-young-adult; DTC, differentiated thyroid cancer; ECM, extracellular matrix; EMT, epithelial-mesenchymal transition; FTC, follicular thyroid carcinoma; HGFCDTC, high-grade follicular-cell-derived thyroid carcinoma; NIFTP, noninvasive follicular thyroid neoplasm with papillary-like nuclear features; PDTC, poorly differentiated thyroid carcinoma; PTC, papillary thyroid carcinoma; RAI, radioactive iodine; TAM, tumor-associated macrophage; TLS, tertiary lymphoid structure.

## Cell-cell signaling and therapeutic hypotheses

The translational value of a candidate cell-cell interaction depends on the evidence level and the cell type in which the target is expressed. Transcript-inferred ligand-receptor pairs are useful for nominating hypotheses, but drug targeting requires more than transcript co-expression. Spatial colocalization strengthens the case by placing the source and target cells in the same tissue region. Protein-level validation confirms that the relevant molecules are present. Perturbation in co-culture, organoid, xenograft or immunocompetent models tests dependency, and clinical or treatment-linked cohorts are needed before these candidates can guide patient selection. This distinction is especially important in ATC, where tumor cells, macrophages, CAFs, endothelial cells and T cells may each provide different therapeutic entry points (19).

**Figure 3:**
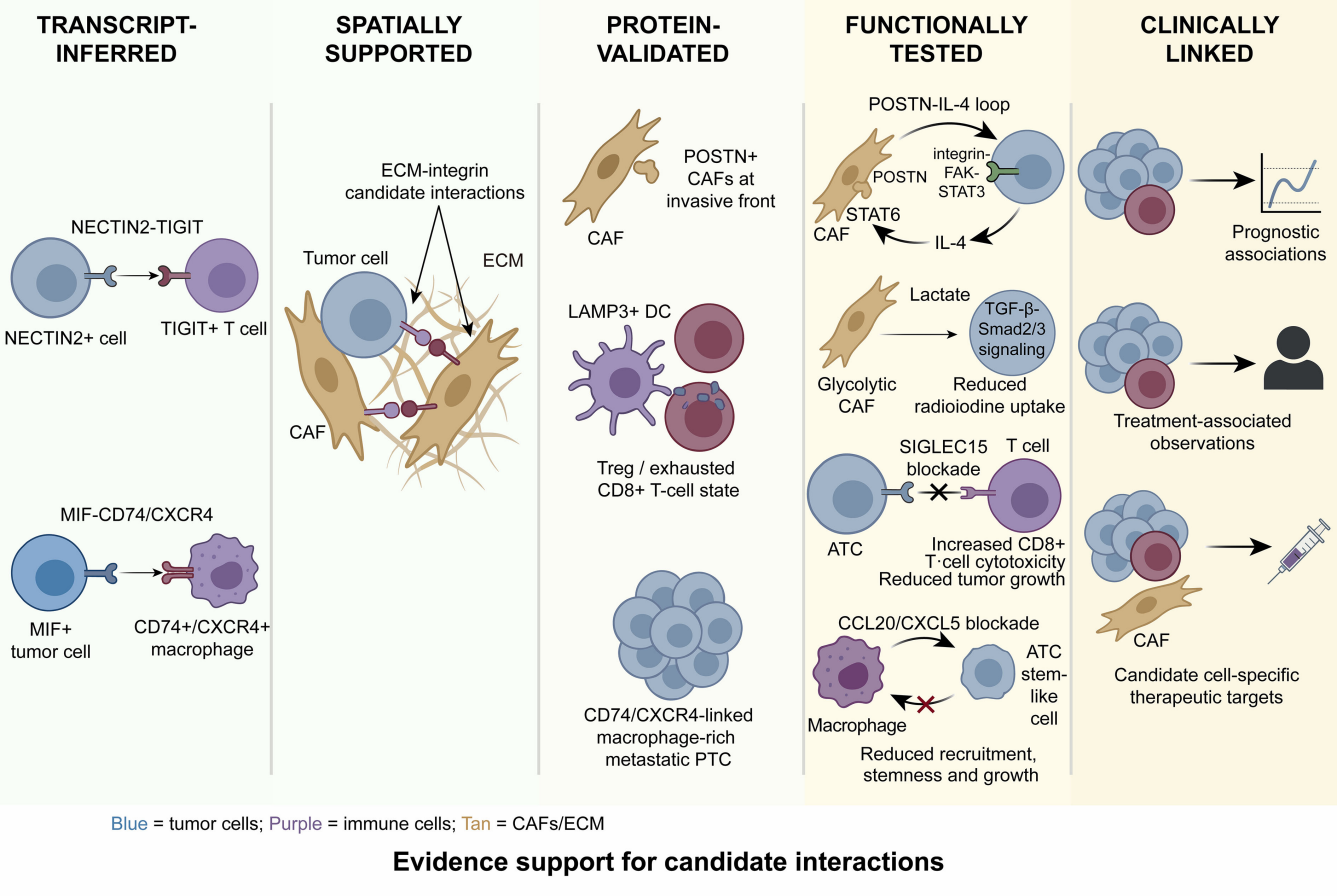
Evidence support for candidate intercellular communication axes. The schematic separates candidate interactions by the type of support available: transcript-level inference, spatial localization, protein validation, functional testing and clinical linkage. Examples include NECTIN2-TIGIT and MIF-CD74/CXCR4 as transcript-inferred candidates, ECM-integrin interactions as spatially supported candidates, POSTN+ CAFs, LAMP3+ DC-associated Treg or exhausted CD8+ T-cell states, and CD74/CXCR4-linked macrophage-rich metastatic PTC as protein-supported observations, and POSTN-IL-4, CAF lactate/TGF-beta-Smad2/3, SIGLEC15 blockade and CCL20/CXCL5 blockade as functionally tested examples. The clinically linked column denotes prognostic associations, treatment-associated observations and candidate cell-specific therapeutic targets.

In thyroid cancer, intercellular signaling analyses link malignant epithelial states to neighboring stromal and immune cell populations. Ligand-receptor pairs are most informative when supported by disease context, spatial localization, perturbation experiments or treatment-associated samples. Many interactions remain computationally inferred; a smaller group has support from co-culture systems, animal models, spatial assays or treated tumors. These analyses depend on resources such as CellPhoneDB, NicheNet and related tumor communication workflows, whose assumptions influence inferred pairs and prioritized ligands (69–71). Current evidence highlights four recurring signaling settings: tumor-immune suppression in progressive PTC, macrophage and checkpoint-related interactions in ATC, CAF-tumor signaling linked to invasion or dedifferentiation, and lymphoid organization associated with tumor restraint or immunotherapy sensitivity. **Figure 3** summarizes the evidence categories used to interpret candidate interactions. **Table 2** summarizes cell type-specific interactions and therapeutic targets, with evidence support, translational relevance and current limitations.

**Table 2:** Translational implications of candidate cell type-specific interactions and therapeutic targets.

Disease setting and candidate target/interaction	Source and target cells	Evidence support	Translational relevance and evidence limits	PMID
Progressive PTC; NECTIN2-TIGIT and CCL17-CCR4	LAMP3+ DCs, epithelial/endothelial cells, exhausted CD8+ T cells and Tregs	scRNA-seq plus spatial/protein validation; limited perturbation	Candidate immunosuppressive signals for stratification and future immunomodulatory testing; not yet treatment biomarkers.	38816233; 41249602
Metastatic PTC; MIF-CD74/CXCR4	Malignant epithelial or stromal/immune cells to macrophages	scRNA-seq communication analysis plus clinical validation	May mark macrophage suppression in metastatic PTC; functional targeting remains unproven.	38061122; 39351536
PTC invasive stroma; POSTN-IL-4 reciprocal signaling	POSTN+ CAFs and tumor cells	Functional PTC models and signaling assays	Supports CAF-tumor signaling as a possible stromal vulnerability; no patient-selection assay or trial evidence.	38773979
PTC, PDTC and ATC regions; CAF lactate/TGF-beta-Smad2/3	Glycolytic CAFs to tumor cells	ST plus conditioned-medium and co-culture assays	Links stromal metabolism with invasion, dedifferentiation and reduced RAI uptake; *in vivo* and clinical validation needed.	41761234
ATC stem-like/macrophage-rich tumors; CCL20/CXCL5 reciprocal signal	M2-like macrophages and ATC stem-like cells	Cytokine arrays, proteomics, co-culture and orthotopic ATC models	Functionally supported TAM-stem-like tumor-cell interaction; clinical translation and druggability remain unresolved.	40091357
DTC-to-ATC dedifferentiation; TYMP+ TAM signal	ATC-region macrophages and tumor regions	ST, WES/inferCNV and validation analyses	Connects ATC regions with macrophage-rich suppression; therapeutic dependency still needs testing.	40908584
ATC; SIGLEC15 checkpoint signaling	SIGLEC15-high tumor cells and T cells	Co-culture, zebrafish xenografts and murine ATC models	Candidate checkpoint target in ATC, potentially complementing PD-1/PD-L1 strategies; human validation needed.	38652971
Aggressive thyroid cancer/ATC-related models; VSIG4+ TAM and SPP1-linked myeloid program	VSIG4+ TAMs, neutrophil-linked myeloid programs and CD8+ T cells	Functional blockade and antigen-presentation assays	Candidate myeloid-directed combination strategy for myeloid-rich aggressive tumors; evidence is not thyroid-cancer-exclusive.	39920772
BRAF V600E-mutant ATC; MAPK reactivation, EphA2 signaling and CD163+ TAM enrichment	Tumor cells plus macrophage-rich microenvironment	Clinical targeted-therapy activity, retrospective data and multi-region resistance profiling	Supports rapid molecular profiling and resistance-informed combinations; evidence is context dependent and mostly non-randomized.	35026411; 37059834; 38226606; 38990526; 39395384; 42143022
ATC and dedifferentiated thyroid cancer; epigenetic regulators including HDAC/EZH2-linked differentiation programs	Tumor-cell chromatin and differentiation programs, with downstream immune/TME effects	Preclinical and early translational evidence	Rationale for combining MAPK inhibition with epigenetic/redifferentiation strategies; evidence is heterogeneous and not practice-changing.	40819127; 30842140; 36854530; 37921808
Indolent early-stage PTC; B-cell/TLS-related interactions such as PTPRC-CD22	Tumor-infiltrating B cells, germinal-center B cells and TLS-like structures	scRNA-seq/ST observations plus B-cell functional testing	May distinguish lymphoid-organized restrained tumors from progressive immune suppression; mainly a stratification hypothesis.	40069827
Pediatric thyroid carcinoma; ITGA2-JAK-STAT3 macrophage polarization	ITGA2-high tumor cells to macrophages	Pediatric organoid, *in vivo* validation and multiplex IHC	Pediatric-specific metabolic-immune interaction; adult generalization is limited.	40985182

Evidence support is summarized qualitatively to distinguish transcript-level inference, spatial localization, protein validation, functional testing and clinical or treatment-associated observations. These axes should be interpreted as translational hypotheses rather than established clinical biomarkers. ATC, anaplastic thyroid carcinoma; CAF, cancer-associated fibroblast; DTC, differentiated thyroid cancer; HDAC, histone deacetylase; MAPK, mitogen-activated protein kinase; PDTC, poorly differentiated thyroid carcinoma; PTC, papillary thyroid carcinoma; RAI, radioactive iodine; TAM, tumor-associated macrophage; TLS, tertiary lymphoid structure.

In progressive and metastatic PTC, several studies converge on tumor-immune suppression. The clearest example is the interaction between LAMP3+ dendritic cells and T-cell populations in progressive PTC. In this study, LAMP3+ dendritic cells were more common in progressive tumors and were linked to CD8+ T-cell exhaustion through NECTIN2-TIGIT signaling and to Treg recruitment through CCL17-CCR4 signaling; tumor cells were also proposed to retain LAMP3+ dendritic cells through NECTIN3-NECTIN2 interactions (55). An independent transcriptomic reanalysis also highlighted NECTIN-TIGIT signaling as a candidate contributor to T-cell exhaustion in PTC, with NECTIN2/3 signals in epithelial or endothelial cells and TIGIT expression in exhausted T cells (72). Tumor-macrophage communication adds another layer to metastatic PTC. In paired primary, adjacent and lymph-node metastatic samples, malignant epithelial cells and macrophages formed strong inferred communication, with MIF-(CD74+CXCR4) identified as the top epithelial-macrophage ligand-receptor pair and CD74 higher in metastatic tumors (57). Thus, progressive or metastatic PTC can contain coordinated dendritic-cell, T-cell and macrophage signals that reduce antitumor immunity.

In ATC, communication studies point more strongly toward macrophage-rich and stem-like tumor-cell states. A study integrating single-cell RNA sequencing, cytokine and chemokine arrays, proteomics, co-culture assays and orthotopic ATC stem-cell models identified a CCL20/CXCL5 reciprocal signal between tumor-associated macrophages and anaplastic thyroid cancer stem cells (73). M2-like macrophage-derived CCL20 activated IRAK1/NF-kB signaling in ATC stem-like cells, while CXCL5 secreted by these cells supported stemness and promoted macrophage recruitment and M2 polarization. Spatial transcriptomics of tumors containing coexisting DTC, PDTC and ATC regions identified another ATC-associated macrophage signal: TYMP+ TAMs were concentrated in ATC regions, and loss of PDCD4 promoted immunosuppressive effectors and TYMP+ TAM infiltration (17). A separate ATC study nominated SIGLEC15 as a tumor-cell checkpoint target. SIGLEC15high ATC cells interacted with T cells through immunosuppressive signals including MIF-TNFRSF14 and CXCL12-CXCR4, and anti-SIGLEC15 antibody increased CD8+ T-cell cytotoxicity in co-culture, zebrafish-derived xenografts and immunocompetent murine ATC models (74). A broader study of aggressive cancers, including ATC models, further showed that VSIG4+ TAM blockade restored antigen-presentation signals and improved antigen-specific CD8+ T-cell activity through an SPP1-linked myeloid-neutrophil program (75). Functional assays in these studies strengthen the biological rationale for macrophage- and checkpoint-directed combinations in ATC.

BRAF-mutant ATC illustrates how tumor-intrinsic and microenvironmental resistance can converge. Dabrafenib plus trametinib has established activity in BRAFV600E-mutant ATC (76, 77). Consensus recommendations now emphasize rapid molecular profiling and timely BRAF/MEK-directed treatment (78). However, many patients still develop adaptive or acquired resistance. Retrospective clinical data suggest that adding PD-1 blockade or using neoadjuvant targeted therapy may improve outcomes in selected patients, but these data remain non-randomized and treatment-context dependent (79, 80). Mechanistic studies indicate that resistance involves more than simple failure of drug exposure. A recent multi-region WGS/WES and single-nucleus RNA-seq study of RAF/MEK inhibitor-treated ATC identified MAPK pathway reactivation together with expansion of CD163+ tumor-associated macrophages in a resistant tumor. In cell-line and patient-derived xenograft models, the type II RAF inhibitor naporafenib, especially with trametinib, suppressed EphA2-associated MAPK and PI3K-AKT signaling and overcame innate or acquired resistance to type I RAF/MEK inhibition; compensatory MAST1 alterations were proposed as one mechanism of resistance to naporafenib-based treatment (81). A separate vemurafenib-resistant ATC cell-line study implicated YAP-associated HER3 activation and downstream MAPK/PI3K signaling, with altered STAT3 and stemness behavior (82). Together, these findings suggest that resistance in BRAF-mutant ATC can involve tumor-cell pathway rewiring and a macrophage-rich suppressive microenvironment. Combination strategies pairing MAPK inhibition with immune, myeloid-cell or next-generation RAF-directed approaches are biologically plausible, but they should be presented as trial-level or preclinical hypotheses until validated prospectively.

Epigenetic regulation provides another link between oncogenic signaling, thyroid-identity loss and treatment resistance. DNA methylation, histone modification, chromatin remodeling and chromatin accessibility can alter thyroid differentiation genes and may influence whether MAPK inhibition restores or fails to restore thyroid differentiation programs. This is relevant to ATC because loss of differentiation and immune remodeling occur together with genomic instability and altered chromatin states. HDAC inhibitors, EZH2 inhibition and strategies that modify NIS trafficking or radioiodide uptake have shown preclinical or early translational activity in thyroid cancer models, but the evidence remains heterogeneous. For example, EZH2 inhibition suppressed growth in ATC cell lines without restoring thyroid differentiation markers, whereas vorinostat combined with chloroquine enhanced radionuclide uptake in experimental systems by targeting NIS endocytosis. At present, MAPK plus epigenetic or redifferentiation-oriented combinations remain testable therapeutic hypotheses (83–86).

CAF-tumor signaling links the stromal regions described above to malignant behavior. CAF-derived periostin promoted PTC growth through integrin-FAK-STAT3 signaling and induced tumor-cell IL-4 expression; tumor-derived IL-4, in turn, activated CAFs and stimulated POSTN expression through STAT6 (44). This reciprocal POSTN-IL-4 signal is one of the more experimentally supported CAF-tumor interactions in PTC. Dedifferentiation-associated CAF metabolism provides a second example. Spatial transcriptomics across PTC, PDTC and ATC regions found increased CAF abundance and glycolytic activity in poorly differentiated areas; CAF-conditioned medium promoted PTC proliferation, invasion and dedifferentiation and reduced radioiodine uptake, while CAF-secreted lactate activated TGF-beta/Smad2/3 signaling (45). Spatial studies also report candidate matrix-related ligand-receptor interactions, including LAMB3-ITGA2, FN1-ITGA3 and FN1-SDC4 in PTC transition regions (87). These matrix-related interactions remain spatial candidates, whereas POSTN-IL-4 and CAF-lactate/TGF-beta have direct experimental support.

Lymphoid organization represents a different class of interaction from the immune-suppression signals described above. In early-stage PTC, tumor-infiltrating B cells, especially germinal-center B cells, were more abundant in indolent tumors, formed TLS-like clusters and suppressed thyroid-cell proliferation in validation assays (60). The same study identified PTPRC-CD22 interactions as potential drivers of TIL-B-cell proliferation, suggesting that B-cell organization may help distinguish tumors suitable for surveillance from those more likely to progress. In ATC, CXCL13+ T cells and early TLS-like structures were more abundant in tumor samples and may contribute to the greater immunotherapy sensitivity of ATC compared with late-stage PTC; famitinib plus anti-PD-1 advanced TLS-related signals in murine experiments and in a treated patient sample (64). These findings show that intercellular signaling can also mark organized lymphoid responses. Spatially organized lymphoid signals may indicate a different therapeutic state from diffuse myeloid-rich suppression.

Several newer studies add candidate interactions outside the main adult PTC-ATC progression evidence. Papillary thyroid microcarcinoma analysis has nominated PROS1-MERTK signaling as a candidate link between thyrocytes, macrophage programs and local progression (88). CXCL8+ monocytes may interact with SDC1+ tumor stem cells to activate JAK-STAT signaling and promote thyroid cancer stem-cell self-renewal, invasion and distant metastasis, although the disease context is broad thyroid cancer and includes metastatic material beyond typical PTC cohorts (89). Stemness-high PTC cells may communicate with endothelial cells through JAG1-NOTCH1/4 signaling, but this remains mainly a computational finding linked to TCGA survival analysis (90). Pediatric PTC provides another context in which ITGA2high tumor cells appear to promote M2 macrophage polarization through JAK-STAT3 signaling (91). Their relevance to adult PTC-to-ATC progression remains secondary until stronger validation or broader cohorts are available.

Functionally tested interactions currently provide the clearest translational leads. The most actionable candidates include ATCSC-TAM CCL20/CXCL5 signaling, SIGLEC15 blockade in ATC models, VSIG4+ TAM targeting in ATC-related models, CAF-derived POSTN/IL-4 signaling and CAF lactate-driven dedifferentiation. NECTIN-TIGIT, MIF-CD74/CXCR4, PTPRC-CD22, JAG1-NOTCH and spatial ECM-integrin signals remain important candidates, but most require direct perturbation, protein-level validation and clinical correlation before they can guide treatment selection. The current evidence links metastatic PTC with dendritic-cell, T-cell and macrophage suppression; dedifferentiated ATC with macrophage-rich and checkpoint-related signaling; stromal-rich regions with CAF-tumor signaling; and lymphoid-organized tumors with potential immune responsiveness.

## Limitations and future directions

Current studies describe many cellular associations, but only a small fraction has tested whether these associations drive invasion, dedifferentiation or treatment response. Single-cell and spatial transcriptomic studies have identified malignant epithelial states, stromal regions and immune patterns across thyroid cancer progression, but most datasets infer progression from cross-sectional cohorts, disease-state comparisons or spatially adjacent regions. This limits conclusions about temporal order, functional dependence and treatment relevance. It also constrains subtype-level conclusions. The strongest evidence comes from PTC, DTC-to-ATC transformation and ATC (13–15). FTC, PDTC, MTC and dedicated RAI-refractory cohorts remain sparsely sampled (49, 92, 93).

This association-rich evidence base partly reflects the strengths and limitations of the technologies themselves. Dissociated scRNA-seq resolves rare or transitional cell states but loses native tissue context, while spatial transcriptomics preserves location but varies in resolution, gene coverage, segmentation performance and platform sensitivity (94, 95). Benchmarking studies show that spatial deconvolution, transcript distribution prediction and clustering can vary substantially across methods (96, 97). Spatially resolved communication methods address some proximity questions but also introduce additional modeling choices (98). Ligand-receptor tools such as CellChat and LIANA+ can rank candidate interactions and compare signaling patterns across cell states (99, 100). Comparative and method-development studies also show that co-expression of a ligand and receptor does not by itself establish active signaling, directionality or therapeutic dependency (101–103). A practical way to handle these candidate interactions is to grade them by evidence level: transcript-inferred, spatially supported, protein-validated, functionally tested or clinically linked.

Future studies need to move from describing associations to testing functional dependencies. Priority designs include matched multi-region or serial sampling across primary tumors, lymph-node metastases, distant or RAI-refractory lesions and anaplastic transformation; spatial multi-omics that pairs spatial transcriptomic data with metabolite, protein or matrix readouts; spatial validation of small regions such as invasive fronts, transition zones and lymphoid aggregates; and perturbation studies in co-culture, organoid, animal or patient-derived systems. Initial thyroid cancer examples have already linked spatial transcriptomics to metabolomic or metabolic validation (45, 104). Functional studies of CAF-tumor and macrophage-tumor signaling show that this step is feasible (44, 73). Checkpoint-focused and pediatric organoid-based studies provide additional examples of how cellular-state findings can be connected to perturbation or model validation (74, 91).

At this stage, single-cell and spatial transcriptomics are most useful for identifying where mechanisms should be tested. Their clinical value will depend on studies that determine which cellular states and interactions are reproducible, functionally required and measurable in specimens suitable for risk stratification or treatment selection.

## Conclusions

Single-cell and spatial transcriptomic studies have changed thyroid cancer research from a marker-centered description of cell populations to a tissue-level view of malignant states, stromal regions and immune patterns. The most consistent evidence links progression with loss of differentiated thyroid identity, partial EMT or ATC-like malignant states, CAF/ECM-rich invasive and poorly differentiated regions, myeloid and T-cell suppressive immune states, and a smaller group of experimentally tested cell-cell interactions. The next step is to determine which of these associations are reproducible, functionally required and measurable in routine or trial-linked specimens.

## Supplementary material

Supplementary Table 1 Original single-cell, spatial and functional studies supporting the review.
