# Supplementary material for: Cell-state transitions and microenvironmental remodeling in thyroid cancer progression revealed by single-cell and spatial transcriptomics
Source: Front Immunol. 2026 Jul 8;17:1904196. doi: 10.3389/fimmu.2026.1904196 (PMC13388755; doi:10.3389/fimmu.2026.1904196)
Supplement: Supplementary Table 1 — Original single-cell, spatial and functional studies supporting the review. [file Table1.docx]

**Supplementary Table S1. Original single-cell, spatial and functional studies supporting the review**

PMIDs are retained as plain identifiers for EndNote checking and reference insertion.

| **Evidence topic** | **Disease setting and method** | **Key cell state or region** | **Main relevance and evidence limit** | **PMID** |
| --- | --- | --- | --- | --- |
| PTC clinical-course cell states | PTC, paratumor tissue, lymph-node metastasis and RAI-refractory distant metastasis; 11 patients; scRNA-seq | Cancer-primed thyrocytes; follicular-like, partial EMT-like and dedifferentiation-like malignant cells | Supports malignant-cell state shifts linked to subtype, prognosis and RAI response; mainly cross-sectional. | 34663816 |
| DTC-to-ATC transformation | Differentiated to anaplastic thyroid tumors; scRNA-seq plus genetic analysis | Stress-responsive DTC, inflammatory/mesenchymal ATC, CAFs, macrophages and exhausted T cells | Supports thyroid-identity loss and inflammatory/mesenchymal ATC states; longitudinal human validation is limited. | 37053016 |
| Integrated stromal and spatial study | WDTC, ATC and pediatric thyroid carcinoma; 81 samples and 28 spatially profiled tumors; scRNA-seq, ST and bulk validation | POSTN+ myCAF, APOD+ inflammatory CAF and invasive tumor cells | Links POSTN+ myCAF with invasive borders, metastasis and outcome; CAF function is mainly inferred outside selected experiments. | 41480746 |
| Spatial progression across disease states | Paratumor thyroid, PTC, locally advanced PTC and ATC; ST with scRNA-seq integration | Leading-edge regions, SERPINE1+ fibroblasts and stage-associated tumor/stromal regions | Shows region-specific tumor and stromal changes with progression; interaction and prognostic claims need cautious interpretation. | 40157360 |
| Spatial dedifferentiation and ATC regions | Coexisting DTC, PDTC and ATC regions in seven tumors; ST, WES, inferCNV and validation assays | Low-differentiation regions, SERPINE1+/KRT5+ ATC regions and TYMP+ TAMs | Links dedifferentiation with immune-suppressive, angiogenic and ECM-rich ATC regions; sample number is small. | 40908584 |
| TME across differentiation states | PTC, FTC, PDTC and ATC from 12 patients; FFPE ST and IHC | Fibroblast-rich and myeloid-rich low-differentiation regions | Lower thyroid differentiation score associates with CAF and myeloid enrichment; subtype conclusions need larger cohorts. | 41182416 |
| Metastasis-associated malignant state | Advanced PTC and lymph-node metastasis; scRNA-seq, ST and functional assays | APOE-low or APOE-negative tumor cells | Links APOE-low/negative cells with advanced stage and nodal metastasis; broader specificity needs validation. | 39810624 |
| PTC partial EMT program | Human PTC and clinical outcome cohorts; scRNA-seq reanalysis, consensus NMF and cell assays | EMT-related malignant program and ELF3-associated GEP3-high cells | Supports recurrent partial EMT in metastatic or poor-outcome PTC; depends partly on public datasets. | 40048012 |
| BRAF-driven PTC plasticity | Adult-onset BRAF V600E mouse PTC model; scRNA-seq, trajectory analysis and organoids | Malignant thyrocyte subpopulations along an EMT trajectory | Shows BRAF-mutant PTC can contain intermediate and mesenchymal states; human extrapolation needs caution. | 41935217 |
| RAI-refractory dedifferentiation | BRAF-mutated RAI-refractory PTC cells; mechanistic molecular assays | NIS suppression and PAX8/NKX2.1 chromatin-access impairment | Links RAI resistance to reduced iodide-handling gene expression; patient-level validation remains needed. | 41694580 |
| Dedifferentiation and DNA-damage response | Thyroid cancer and adjacent normal tissues; public scRNA-seq plus experimental validation | ATM-high dedifferentiation-associated malignant cells | Suggests DNA-damage-response programs accompany dedifferentiation and RAI resistance; prospective validation is needed. | 42046098 |
| Relapsed FTC and ATC-like cells | PTC, FVPTC, relapsed FTC and ATC; scRNA-seq and functional assays | UBE2C-high ATC-like FTC cells | Shows ATC-like states can appear beyond PTC-derived transformation; FTC evidence is small. | 41631714 |
| PTC invasive-front fibrosis | Large PTC pathology cohort plus single-cell analysis; pathomics, scRNA-seq and in vitro assays | Metastasis-associated myoCAFs and CD36+ CAFs | Links invasive-front fibrosis and CAF states with lymph-node metastasis; spatial transcriptomic resolution is limited. | 38858612 |
| Peritumoral stiffness | Large clinical PTC cohort; elastography, multiplex IF and molecular assays | Stiff peritumoral matrix with alpha-SMA, PDGFR-alpha, p-MLC2 and COL-I regions | Links matrix stiffness with nodal metastasis, recurrence and mortality; patient-level mechanism remains incomplete. | 38902966 |
| ATC stiffness response | ATC model systems; hydrogel stiffness assays and signaling inhibition | Integrin alpha6beta4-FAK activity under high stiffness | Shows stiffness can increase ATC proliferation, migration and invasion; patient-level spatial confirmation is needed. | 41606295 |
| FTC invasive regions | One follicular thyroid carcinoma case plus IHC cohort; ST and IHC | CD74-high invasive/peripheral tumor subpopulation, POSTN and DPYSL3 peripheral signals | Provides an FTC invasion example with spatially restricted regions; this is a single-case discovery. | 38280140 |
| Progressive PTC immune suppression | Adjacent tissue, non-progressive PTC and progressive PTC; 18 specimens; scRNA-seq, ST, IHC, multiplex IHC and bulk validation | LAMP3+ dendritic cells, exhausted CD8+ T cells and Tregs | Links progressive PTC to antigen-presentation dysfunction and T-cell suppression; perturbation evidence is still needed. | 38816233 |
| Metastatic PTC macrophage communication | Primary PTC, adjacent thyroid and lymph-node metastasis; scRNA-seq, communication analysis and TCGA validation | Macrophages and epithelial tumor cells | Suggests MIF-CD74/CXCR4-related macrophage suppression in metastatic PTC; functional targeting remains unproven. | 38061122 |
| Metastatic PTC TME | Metastatic PTC, adjacent tissue and non-metastatic PTC; scRNA-seq | M2-like macrophages, cDC2, Tregs, monocytes and B cells | Associates metastasis with myeloid and regulatory immune states; treatment implications remain exploratory. | 40496475 |
| Indolent PTC immune state | Early-stage PTC with validation cohort; scRNA-seq, co-culture, IF and flow cytometry | Tumor-infiltrating B cells, germinal-center B cells and TLS-like clusters | Shows that immune infiltration can accompany tumor restraint; focused on indolent early-stage PTC. | 40069827 |
| ATC immune suppression | PTC and ATC tissues; scRNA-seq and mIHC validation | Exhausted CD8+ T cells, M2 macrophages and checkpoint programs | Supports a myeloid-rich and T-cell-dysfunctional ATC immune state; ATC sample size is limited. | 37679527 |
| ATC immunotherapy-related lymphoid signals | PTC and ATC with treatment-associated context; scRNA-seq and validation | CXCL13+ T cells and early TLS-like structures | Links organized lymphoid signals with immunotherapy sensitivity in selected ATC settings; context is important. | 38478516 |
| ATC pre-exhausted CD8+ T cells | Public ATC and PTC single-cell datasets with experimental validation | Pre-exhausted CD8+ T cells and GNLY-associated program | Reinforces CD8+ dysfunction in ATC; candidate biomarkers require clinical validation. | 39752183 |
| Large thyroid cancer TME landscape | 405,077 cells from thyroid cancer and normal thyroid samples; integrated scRNA-seq plus mIHC validation | APOE+ macrophages, EMT-like CAFs and RBP7+ endothelial cells | Provides broad context for immune, stromal and endothelial variation; should not dominate subtype-specific claims. | 41298873 |
| HT-associated immune context | HT-associated PTC and related thyroid tissues; scRNA-seq, CNV inference and validation analyses | B cells, plasma cells, macrophages, mast-cell and inflammatory programs | Shows autoimmune thyroiditis modifies the immune context of PTC; HT-specific findings should not be generalized. | 34805166; 39351536; 41808291 |
